# 4′-(2,4-Dichloro­phen­yl)-1,1′-dimethyl­piperidine-3-spiro-3′-pyrrolidine-2′-spiro-3′′-indoline-4,2′′-dione

**DOI:** 10.1107/S160053681000704X

**Published:** 2010-02-27

**Authors:** S. Nagamuthu, R. Sribala, R. Ranjithkumar, R. V. Krishnakumar, N. Srinivasan

**Affiliations:** a Department of Physics, Thiagarajar College, Madurai 625 009, India; b School of Chemistry, Madurai Kamaraj University, Madurai 625 021, India

## Abstract

In the title compound, C_23_H_23_Cl_2_N_3_O_2_, the pyrroline ring adopts an envelope conformation and the piperidinone ring assumes a slightly twisted chair form. In the crystal, inversion dimers linked by pairs of N—H⋯O hydrogen bonds generate an *R*_2_^8^ graph-set motif and a short Cl⋯Cl contact of 3.478 (1) Å occurs.

## Related literature

For the effect on halogens on the conformations of organic mol­ecules, see: Awwadi *et al.* (2006 ▶). For the biological properties of pyrroles, see: Watson *et al.* (2001 ▶). For graph-set notation, see: Etter *et al.* (1990 ▶). For puckering parameters, see: Cremer & Pople (1975 ▶). 
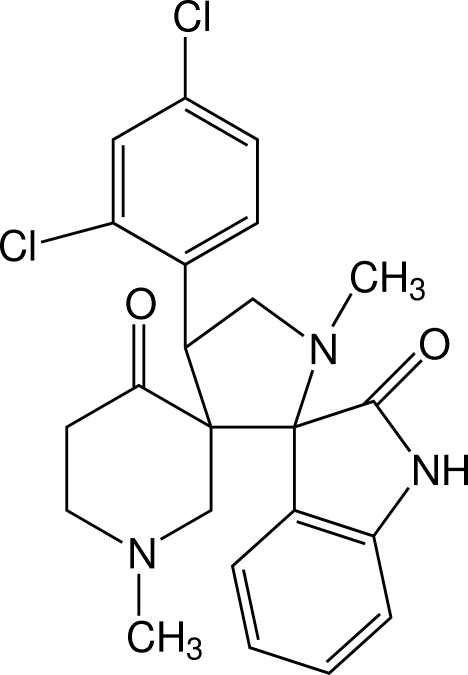


## Experimental

### 

#### Crystal data

C_23_H_23_Cl_2_N_3_O_2_
*M* *_r_* = 444.34
Triclinic, 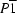

*a* = 7.9398 (2) Å
*b* = 10.8747 (3) Å
*c* = 13.5367 (4) Å
α = 66.561 (2)°
β = 77.873 (1)°
γ = 83.203 (2)°
*V* = 1047.64 (5) Å^3^
*Z* = 2
Mo *K*α radiation
μ = 0.34 mm^−1^
*T* = 300 K
0.27 × 0.15 × 0.12 mm

#### Data collection

Bruker Kappa APEXII CCD diffractometer
Absorption correction: multi-scan (*SADABS*; Sheldrick, 1996 ▶) *T*_min_ = 0.94, *T*_max_ = 0.96
27365 measured reflections
6685 independent reflections
5249 reflections with *I* > 2σ(*I*)
*R*_int_ = 0.025

#### Refinement

*R*[*F* ^2^ > 2σ(*F* ^2^)] = 0.044
*wR*(*F* ^2^) = 0.130
*S* = 1.03
6685 reflections
273 parameters
H-atom parameters constrained
Δρ_max_ = 0.47 e Å^−3^
Δρ_min_ = −0.47 e Å^−3^

### 

Data collection: *APEX2* (Bruker, 2004 ▶); cell refinement: *SAINT* (Bruker, 2004 ▶); data reduction: *SAINT*; program(s) used to solve structure: *SHELXS97* (Sheldrick, 2008 ▶); program(s) used to refine structure: *SHELXL97* (Sheldrick, 2008 ▶); molecular graphics: *PLATON* (Spek, 2009 ▶); software used to prepare material for publication: *SHELXL97*.

## Supplementary Material

Crystal structure: contains datablocks I, global. DOI: 10.1107/S160053681000704X/ng2738sup1.cif

Structure factors: contains datablocks I. DOI: 10.1107/S160053681000704X/ng2738Isup2.hkl

Additional supplementary materials:  crystallographic information; 3D view; checkCIF report

## Figures and Tables

**Table 1:** Hydrogen-bond geometry (Å, °)

*D*—H⋯*A*	*D*—H	H⋯*A*	*D*⋯*A*	*D*—H⋯*A*
N1*B*—H1*B*⋯O2^i^	0.86	2.02	2.8547 (15)	164

Symmetry code: (i) 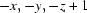.

**Fig. 1.**
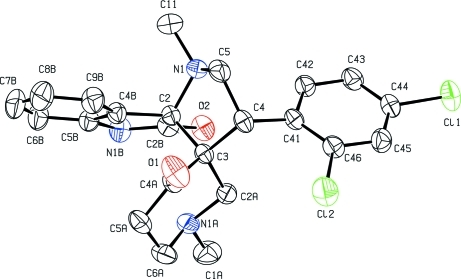
The molecular structure of (I), with atom labels and 50% probability displacement ellipsoids for non-H atoms. H atoms have been omitted for clarity.

**Fig. 2.**
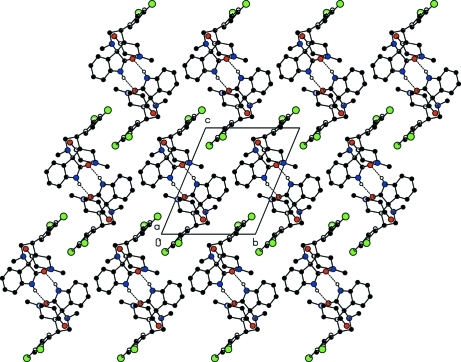
A view of the molecular aggregation down the a-axis. C-bound H atoms have been omitted for clarity.

## supplementary crystallographic information

### Comment

Pyrrolo ring compounds are prevalent in a variety of biologically active compounds (Watson *et al.*, 2001) and find utility in the treatment of diseases such as diabetes, cancer and viral infections. Since the biological activity depends on the conformation of rings that constitute a molecule, precise description, at atomic resolution, of the title compound is expected to supplement further studies on structure-activity relationships on these compounds. Also, the effect of halogen- subsitutions on the conformation of the molecule and consequently on the packing modes remains an area of immense interest in crystallography (Awwadi *et al.*, 2006).

In the title compound (I), the 5-membered methyl substitued pyrroline ring adopts the envelope conformation with C5 deviating from the plane defined by the rest of the atoms of the ring by 0.639 (2) A° The puckering parameters (Cremer & Pople, 1975) of this ring are Q = 0.431 (2)Å and φ = 331.2 °. The piperidinone ring adopts a slightly twisted chair conformation with the N1A and C4A atoms deviating by about 0.712 (2) and -0.523 (2) A°, respectively, from the plane defined by C2A,C3,C5A and C6A with an r.m.s. deviation of 0.041 A°. The chair conformation is also evident from the corresponding puckering amplitudes [Q=0.561 (2) A°, /q = 16.0 (2)°, /f = 30.8 (6)°]. The oxindole and the dichloro substituted phenyl rings are planar.

A relatively strong intermolecular [N1B—H1B···O2(-x, -y, 1-z)] hydrogen bond relates centrrosymmetric pairs of molecules (Fig.2). These N—H···O hydrogen bonds form R^8^_2_ graph set motifs (Etter *et al.*, 1990) which are interconnected through Cl···Cl interactions [Cl1···.Cl2 (x-1, y, z) 3.478 (1) A°] leading to columns of molecules parallel to the a-axis (Fig.3). These columns resemble a 'ladder-like' arrangement of molecules much similar to the one seen in DNA, except for the twist. These columns of molecules have interactions that are van der Waals in nature.

### Experimental

A mixture of 1-methyl-3-[(*E*)-(2,4-dichlorophenyl)methylidene] tetrahydro-4(1*H*)pyridinone (1 mmol), isatin (1 mmol) and sarcosine (1 mmol) were refluxed in methanol (15 ml) for 4 h. After completion of the reaction (TLC), the mixture was poured into water (30 ml) and the precipitate was filtered off and washed with water to obtain the product as white solid (0.28 g, 85%), m.p. 190–191 °C

### Refinement

H atoms were positioned geometrically and refined using a riding model with C—H = 0.95–0.99 Å and with *U*_iso_(H) = 1.2 (1.5 for methyl groups) times *U*_eq_(C).

### Crystal data

**Table:**

C_23_H_23_Cl_2_N_3_O_2_	*Z* = 2
*M**_r_* = 444.34	*F*(000) = 464
Triclinic, *P*1	*D*_x_ = 1.409 Mg m^−^^3^
Hall symbol: -P 1	Mo *K*α radiation, λ = 0.71073 Å
*a* = 7.9398 (2) Å	Cell parameters from 5536 reflections
*b* = 10.8747 (3) Å	θ = 5.7–61.9°
*c* = 13.5367 (4) Å	µ = 0.34 mm^−^^1^
α = 66.561 (2)°	*T* = 300 K
β = 77.873 (1)°	Needle, colourless
γ = 83.203 (2)°	0.27 × 0.15 × 0.12 mm
*V* = 1047.64 (5) Å^3^	

### Data collection

**Table:**

Bruker Kappa APEXII CCD diffractometer	6685 independent reflections
Radiation source: fine-focus sealed tube	5249 reflections with *I* > 2σ(*I*)
graphite	*R*_int_ = 0.025
ω and φ scan	θ_max_ = 31.1°, θ_min_ = 2.0°
Absorption correction: multi-scan (*SADABS*; Sheldrick, 1996)	*h* = −10→11
*T*_min_ = 0.94, *T*_max_ = 0.96	*k* = −15→15
27365 measured reflections	*l* = −19→19

### Refinement

**Table:**

Refinement on *F*^2^	Primary atom site location: structure-invariant direct methods
Least-squares matrix: full	Secondary atom site location: difference Fourier map
*R*[*F*^2^ > 2σ(*F*^2^)] = 0.044	Hydrogen site location: inferred from neighbouring sites
*wR*(*F*^2^) = 0.130	H-atom parameters constrained
*S* = 1.03	*w* = 1/[σ^2^(*F*_o_^2^) + (0.0652*P*)^2^ + 0.3075*P*] where *P* = (*F*_o_^2^ + 2*F*_c_^2^)/3
6685 reflections	(Δ/σ)_max_ = 0.001
273 parameters	Δρ_max_ = 0.47 e Å^−^^3^
0 restraints	Δρ_min_ = −0.47 e Å^−^^3^

### Special details

**Table:**

Geometry. All esds (except the esd in the dihedral angle between two l.s. planes) are estimated using the full covariance matrix. The cell esds are taken into account individually in the estimation of esds in distances, angles and torsion angles; correlations between esds in cell parameters are only used when they are defined by crystal symmetry. An approximate (isotropic) treatment of cell esds is used for estimating esds involving l.s. planes.
Refinement. Refinement of *F*^2^ against ALL reflections. The weighted *R*-factor wR and goodness of fit *S* are based on *F*^2^, conventional *R*-factors *R* are based on *F*, with *F* set to zero for negative *F*^2^. The threshold expression of *F*^2^ > σ(*F*^2^) is used only for calculating *R*-factors(gt) etc. and is not relevant to the choice of reflections for refinement. *R*-factors based on *F*^2^ are statistically about twice as large as those based on *F*, and *R*- factors based on ALL data will be even larger.

### Fractional atomic coordinates and isotropic or equivalent isotropic displacement parameters (Å^2^)

**Table:**

	*x*	*y*	*z*	*U*_iso_*/*U*_eq_	
Cl1	−0.05606 (6)	0.11850 (5)	−0.16644 (3)	0.05328 (12)	
Cl2	0.52639 (5)	0.22216 (6)	−0.08290 (4)	0.05850 (14)	
O1	0.59398 (16)	0.37748 (14)	0.13813 (10)	0.0564 (3)	
O2	0.01383 (13)	0.08370 (10)	0.35005 (9)	0.0392 (2)	
N1	0.07639 (15)	0.36986 (11)	0.21024 (10)	0.0342 (2)	
N1A	0.40225 (16)	0.01730 (11)	0.32854 (10)	0.0369 (2)	
N1B	0.09730 (16)	0.15644 (12)	0.46891 (9)	0.0372 (3)	
H1B	0.0533	0.0947	0.5293	0.045*	
C1A	0.3736 (3)	−0.12482 (15)	0.36383 (15)	0.0521 (4)	
H7	0.4034	−0.1725	0.4350	0.078*	
H8	0.4440	−0.1588	0.3127	0.078*	
H9	0.2544	−0.1368	0.3671	0.078*	
C2	0.19382 (16)	0.28182 (11)	0.28156 (10)	0.0277 (2)	
C2A	0.35737 (18)	0.09368 (13)	0.22132 (11)	0.0330 (3)	
H1	0.2483	0.0647	0.2182	0.040*	
H2	0.4449	0.0771	0.1657	0.040*	
C2B	0.09267 (16)	0.15968 (12)	0.36880 (10)	0.0312 (2)	
C3	0.34300 (15)	0.24405 (12)	0.19834 (10)	0.0275 (2)	
C4	0.29884 (17)	0.33178 (13)	0.08305 (10)	0.0317 (2)	
H4	0.4047	0.3711	0.0334	0.038*	
C4A	0.51969 (17)	0.28084 (14)	0.20663 (11)	0.0354 (3)	
C4B	0.24667 (17)	0.34208 (13)	0.35377 (11)	0.0321 (2)	
C5	0.1825 (2)	0.44231 (13)	0.10524 (11)	0.0383 (3)	
H5A	0.2488	0.5087	0.1098	0.046*	
H5B	0.1127	0.4863	0.0489	0.046*	
C5A	0.5987 (2)	0.18768 (17)	0.30320 (14)	0.0442 (3)	
H51	0.5450	0.2069	0.3671	0.053*	
H52	0.7204	0.2048	0.2887	0.053*	
C5B	0.18216 (18)	0.26551 (14)	0.46247 (11)	0.0347 (3)	
C6A	0.5790 (2)	0.04095 (17)	0.32825 (14)	0.0475 (4)	
H61	0.6566	0.0149	0.2736	0.057*	
H62	0.6093	−0.0134	0.3992	0.057*	
C6B	0.3263 (2)	0.45973 (15)	0.32860 (14)	0.0451 (3)	
H6B	0.3695	0.5129	0.2563	0.054*	
C7B	0.3409 (3)	0.49719 (19)	0.41348 (18)	0.0583 (5)	
H7B	0.3940	0.5763	0.3976	0.070*	
C8B	0.2777 (3)	0.4188 (2)	0.52044 (17)	0.0591 (5)	
H8B	0.2893	0.4457	0.5758	0.071*	
C9B	0.1972 (2)	0.30098 (19)	0.54744 (14)	0.0496 (4)	
H9B	0.1550	0.2477	0.6198	0.060*	
C11	−0.0503 (2)	0.45116 (18)	0.25448 (16)	0.0506 (4)	
H11A	−0.1084	0.3957	0.3257	0.076*	
H11B	−0.1328	0.4910	0.2068	0.076*	
H11C	0.0066	0.5205	0.2603	0.076*	
C41	0.21203 (16)	0.26281 (13)	0.03025 (10)	0.0303 (2)	
C42	0.03314 (17)	0.25080 (14)	0.05212 (11)	0.0338 (3)	
H42	−0.0324	0.2744	0.1079	0.041*	
C43	−0.04964 (18)	0.20518 (15)	−0.00610 (12)	0.0375 (3)	
H43	−0.1689	0.1990	0.0101	0.045*	
C44	0.04591 (19)	0.16899 (14)	−0.08833 (11)	0.0362 (3)	
C45	0.22301 (19)	0.17398 (16)	−0.11040 (12)	0.0407 (3)	
H45	0.2879	0.1472	−0.1646	0.049*	
C46	0.30248 (17)	0.21960 (15)	−0.05036 (11)	0.0360 (3)	

### Atomic displacement parameters (Å^2^)

**Table:**

	*U*^11^	*U*^22^	*U*^33^	*U*^12^	*U*^13^	*U*^23^
Cl1	0.0568 (2)	0.0684 (3)	0.0460 (2)	−0.01210 (19)	−0.01697 (18)	−0.02727 (19)
Cl2	0.02974 (17)	0.0955 (4)	0.0552 (2)	−0.00686 (19)	0.00060 (15)	−0.0370 (2)
O1	0.0472 (6)	0.0649 (8)	0.0476 (6)	−0.0279 (6)	−0.0104 (5)	−0.0039 (6)
O2	0.0388 (5)	0.0369 (5)	0.0401 (5)	−0.0137 (4)	−0.0071 (4)	−0.0095 (4)
N1	0.0374 (6)	0.0304 (5)	0.0346 (5)	0.0055 (4)	−0.0123 (4)	−0.0115 (4)
N1A	0.0425 (6)	0.0310 (5)	0.0334 (6)	0.0047 (4)	−0.0116 (5)	−0.0077 (4)
N1B	0.0422 (6)	0.0363 (6)	0.0279 (5)	−0.0105 (5)	0.0006 (4)	−0.0080 (4)
C1A	0.0657 (11)	0.0321 (7)	0.0479 (9)	0.0045 (7)	−0.0105 (8)	−0.0058 (6)
C2	0.0312 (5)	0.0245 (5)	0.0260 (5)	−0.0040 (4)	−0.0068 (4)	−0.0068 (4)
C2A	0.0387 (6)	0.0298 (6)	0.0299 (6)	0.0005 (5)	−0.0078 (5)	−0.0107 (5)
C2B	0.0296 (5)	0.0298 (6)	0.0311 (6)	−0.0048 (4)	−0.0041 (4)	−0.0080 (5)
C3	0.0289 (5)	0.0270 (5)	0.0247 (5)	−0.0039 (4)	−0.0063 (4)	−0.0064 (4)
C4	0.0345 (6)	0.0319 (6)	0.0251 (5)	−0.0082 (5)	−0.0074 (4)	−0.0044 (4)
C4A	0.0309 (6)	0.0414 (7)	0.0347 (6)	−0.0059 (5)	−0.0068 (5)	−0.0137 (5)
C4B	0.0360 (6)	0.0301 (6)	0.0318 (6)	−0.0034 (5)	−0.0085 (5)	−0.0118 (5)
C5	0.0516 (8)	0.0264 (6)	0.0342 (6)	−0.0011 (5)	−0.0176 (6)	−0.0042 (5)
C5A	0.0366 (7)	0.0537 (9)	0.0452 (8)	0.0010 (6)	−0.0187 (6)	−0.0171 (7)
C5B	0.0356 (6)	0.0377 (6)	0.0318 (6)	−0.0004 (5)	−0.0058 (5)	−0.0148 (5)
C6A	0.0436 (8)	0.0490 (8)	0.0475 (8)	0.0132 (7)	−0.0194 (7)	−0.0145 (7)
C6B	0.0556 (9)	0.0349 (7)	0.0478 (8)	−0.0114 (6)	−0.0114 (7)	−0.0154 (6)
C7B	0.0708 (12)	0.0480 (9)	0.0728 (12)	−0.0092 (8)	−0.0219 (10)	−0.0343 (9)
C8B	0.0691 (12)	0.0685 (12)	0.0616 (11)	−0.0013 (9)	−0.0188 (9)	−0.0448 (10)
C9B	0.0564 (9)	0.0613 (10)	0.0385 (8)	−0.0003 (8)	−0.0084 (7)	−0.0275 (7)
C11	0.0498 (9)	0.0479 (9)	0.0586 (10)	0.0166 (7)	−0.0174 (7)	−0.0263 (8)
C41	0.0312 (6)	0.0328 (6)	0.0238 (5)	−0.0038 (4)	−0.0070 (4)	−0.0059 (4)
C42	0.0313 (6)	0.0396 (7)	0.0306 (6)	−0.0019 (5)	−0.0057 (5)	−0.0132 (5)
C43	0.0304 (6)	0.0449 (7)	0.0379 (7)	−0.0035 (5)	−0.0085 (5)	−0.0149 (6)
C44	0.0400 (7)	0.0389 (7)	0.0312 (6)	−0.0039 (5)	−0.0127 (5)	−0.0111 (5)
C45	0.0405 (7)	0.0514 (8)	0.0329 (7)	−0.0016 (6)	−0.0050 (5)	−0.0198 (6)
C46	0.0284 (6)	0.0461 (7)	0.0313 (6)	−0.0028 (5)	−0.0036 (5)	−0.0129 (5)

### Geometric parameters (Å, °)

**Table:**

Cl1—C44	1.7326 (14)	C4B—C5B	1.3894 (19)
Cl2—C46	1.7402 (14)	C5—H5A	0.9700
O1—C4A	1.2057 (18)	C5—H5B	0.9700
O2—C2B	1.2226 (16)	C5A—C6A	1.514 (2)
N1—C11	1.4524 (19)	C5A—H51	0.9700
N1—C5	1.4539 (19)	C5A—H52	0.9700
N1—C2	1.4661 (16)	C5B—C9B	1.381 (2)
N1A—C2A	1.4531 (17)	C6A—H61	0.9700
N1A—C6A	1.455 (2)	C6A—H62	0.9700
N1A—C1A	1.456 (2)	C6B—C7B	1.392 (2)
N1B—C2B	1.3498 (17)	C6B—H6B	0.9300
N1B—C5B	1.3966 (18)	C7B—C8B	1.374 (3)
N1B—H1B	0.8600	C7B—H7B	0.9300
C1A—H7	0.9600	C8B—C9B	1.381 (3)
C1A—H8	0.9600	C8B—H8B	0.9300
C1A—H9	0.9600	C9B—H9B	0.9300
C2—C4B	1.5192 (17)	C11—H11A	0.9600
C2—C2B	1.5524 (16)	C11—H11B	0.9600
C2—C3	1.5916 (17)	C11—H11C	0.9600
C2A—C3	1.5329 (17)	C41—C46	1.3879 (19)
C2A—H1	0.9700	C41—C42	1.3995 (17)
C2A—H2	0.9700	C42—C43	1.3801 (19)
C3—C4A	1.5413 (17)	C42—H42	0.9300
C3—C4	1.5604 (17)	C43—C44	1.376 (2)
C4—C5	1.515 (2)	C43—H43	0.9300
C4—C41	1.5180 (17)	C44—C45	1.378 (2)
C4—H4	0.9800	C45—C46	1.384 (2)
C4A—C5A	1.507 (2)	C45—H45	0.9300
C4B—C6B	1.3811 (19)		
			
C11—N1—C5	115.62 (12)	H5A—C5—H5B	109.2
C11—N1—C2	117.09 (12)	C4A—C5A—C6A	113.15 (13)
C5—N1—C2	106.73 (11)	C4A—C5A—H51	108.9
C2A—N1A—C6A	110.22 (12)	C6A—C5A—H51	108.9
C2A—N1A—C1A	111.33 (12)	C4A—C5A—H52	108.9
C6A—N1A—C1A	111.91 (13)	C6A—C5A—H52	108.9
C2B—N1B—C5B	111.76 (11)	H51—C5A—H52	107.8
C2B—N1B—H1B	124.1	C9B—C5B—C4B	122.56 (14)
C5B—N1B—H1B	124.2	C9B—C5B—N1B	127.53 (14)
N1A—C1A—H7	109.5	C4B—C5B—N1B	109.84 (12)
N1A—C1A—H8	109.5	N1A—C6A—C5A	110.31 (12)
H7—C1A—H8	109.5	N1A—C6A—H61	109.6
N1A—C1A—H9	109.5	C5A—C6A—H61	109.6
H7—C1A—H9	109.5	N1A—C6A—H62	109.6
H8—C1A—H9	109.5	C5A—C6A—H62	109.6
N1—C2—C4B	113.50 (10)	H61—C6A—H62	108.1
N1—C2—C2B	108.33 (10)	C4B—C6B—C7B	118.74 (16)
C4B—C2—C2B	101.02 (10)	C4B—C6B—H6B	120.6
N1—C2—C3	102.91 (9)	C7B—C6B—H6B	120.6
C4B—C2—C3	117.36 (10)	C8B—C7B—C6B	120.87 (16)
C2B—C2—C3	113.80 (10)	C8B—C7B—H7B	119.6
N1A—C2A—C3	110.54 (10)	C6B—C7B—H7B	119.6
N1A—C2A—H1	109.5	C7B—C8B—C9B	121.36 (15)
C3—C2A—H1	109.5	C7B—C8B—H8B	119.3
N1A—C2A—H2	109.5	C9B—C8B—H8B	119.3
C3—C2A—H2	109.5	C8B—C9B—C5B	117.24 (16)
H1—C2A—H2	108.1	C8B—C9B—H9B	121.4
O2—C2B—N1B	125.61 (12)	C5B—C9B—H9B	121.4
O2—C2B—C2	125.83 (12)	N1—C11—H11A	109.5
N1B—C2B—C2	108.44 (10)	N1—C11—H11B	109.5
C2A—C3—C4A	106.08 (10)	H11A—C11—H11B	109.5
C2A—C3—C4	112.94 (10)	N1—C11—H11C	109.5
C4A—C3—C4	109.87 (10)	H11A—C11—H11C	109.5
C2A—C3—C2	113.02 (10)	H11B—C11—H11C	109.5
C4A—C3—C2	110.45 (10)	C46—C41—C42	115.70 (12)
C4—C3—C2	104.55 (10)	C46—C41—C4	122.16 (12)
C5—C4—C41	111.19 (11)	C42—C41—C4	121.86 (12)
C5—C4—C3	102.12 (10)	C43—C42—C41	122.42 (13)
C41—C4—C3	116.79 (10)	C43—C42—H42	118.8
C5—C4—H4	108.8	C41—C42—H42	118.8
C41—C4—H4	108.8	C44—C43—C42	119.36 (13)
C3—C4—H4	108.8	C44—C43—H43	120.3
O1—C4A—C5A	121.14 (13)	C42—C43—H43	120.3
O1—C4A—C3	122.06 (13)	C43—C44—C45	120.56 (13)
C5A—C4A—C3	116.78 (11)	C43—C44—Cl1	120.14 (11)
C6B—C4B—C5B	119.22 (13)	C45—C44—Cl1	119.29 (11)
C6B—C4B—C2	131.56 (13)	C44—C45—C46	118.75 (13)
C5B—C4B—C2	108.82 (11)	C44—C45—H45	120.6
N1—C5—C4	102.60 (10)	C46—C45—H45	120.6
N1—C5—H5A	111.2	C45—C46—C41	123.11 (12)
C4—C5—H5A	111.2	C45—C46—Cl2	115.88 (11)
N1—C5—H5B	111.2	C41—C46—Cl2	121.01 (11)
C4—C5—H5B	111.2		
			
C11—N1—C2—C4B	−36.10 (17)	N1—C2—C4B—C5B	115.04 (12)
C5—N1—C2—C4B	95.27 (12)	C2B—C2—C4B—C5B	−0.69 (14)
C11—N1—C2—C2B	75.23 (15)	C3—C2—C4B—C5B	−125.00 (12)
C5—N1—C2—C2B	−153.40 (11)	C11—N1—C5—C4	179.57 (12)
C11—N1—C2—C3	−163.97 (12)	C2—N1—C5—C4	47.39 (13)
C5—N1—C2—C3	−32.60 (12)	C41—C4—C5—N1	84.59 (12)
C6A—N1A—C2A—C3	−69.88 (14)	C3—C4—C5—N1	−40.70 (12)
C1A—N1A—C2A—C3	165.32 (13)	O1—C4A—C5A—C6A	−136.43 (17)
C5B—N1B—C2B—O2	−173.19 (13)	C3—C4A—C5A—C6A	41.83 (19)
C5B—N1B—C2B—C2	3.01 (16)	C6B—C4B—C5B—C9B	−1.3 (2)
N1—C2—C2B—O2	55.31 (17)	C2—C4B—C5B—C9B	−174.82 (14)
C4B—C2—C2B—O2	174.82 (13)	C6B—C4B—C5B—N1B	176.05 (14)
C3—C2—C2B—O2	−58.48 (17)	C2—C4B—C5B—N1B	2.48 (16)
N1—C2—C2B—N1B	−120.88 (12)	C2B—N1B—C5B—C9B	173.59 (15)
C4B—C2—C2B—N1B	−1.37 (13)	C2B—N1B—C5B—C4B	−3.54 (17)
C3—C2—C2B—N1B	125.33 (11)	C2A—N1A—C6A—C5A	61.70 (16)
N1A—C2A—C3—C4A	58.36 (13)	C1A—N1A—C6A—C5A	−173.83 (14)
N1A—C2A—C3—C4	178.76 (10)	C4A—C5A—C6A—N1A	−47.18 (19)
N1A—C2A—C3—C2	−62.80 (13)	C5B—C4B—C6B—C7B	0.6 (2)
N1—C2—C3—C2A	−117.16 (11)	C2—C4B—C6B—C7B	172.46 (16)
C4B—C2—C3—C2A	117.44 (11)	C4B—C6B—C7B—C8B	0.1 (3)
C2B—C2—C3—C2A	−0.17 (14)	C6B—C7B—C8B—C9B	−0.3 (3)
N1—C2—C3—C4A	124.19 (11)	C7B—C8B—C9B—C5B	−0.3 (3)
C4B—C2—C3—C4A	−1.21 (14)	C4B—C5B—C9B—C8B	1.1 (3)
C2B—C2—C3—C4A	−118.83 (11)	N1B—C5B—C9B—C8B	−175.70 (16)
N1—C2—C3—C4	6.05 (11)	C5—C4—C41—C46	142.59 (13)
C4B—C2—C3—C4	−119.35 (11)	C3—C4—C41—C46	−100.79 (14)
C2B—C2—C3—C4	123.03 (10)	C5—C4—C41—C42	−30.98 (16)
C2A—C3—C4—C5	144.01 (11)	C3—C4—C41—C42	85.64 (15)
C4A—C3—C4—C5	−97.78 (12)	C46—C41—C42—C43	−3.1 (2)
C2—C3—C4—C5	20.75 (12)	C4—C41—C42—C43	170.90 (13)
C2A—C3—C4—C41	22.49 (15)	C41—C42—C43—C44	0.5 (2)
C4A—C3—C4—C41	140.71 (12)	C42—C43—C44—C45	2.0 (2)
C2—C3—C4—C41	−100.77 (12)	C42—C43—C44—Cl1	−177.15 (11)
C2A—C3—C4A—O1	132.58 (15)	C43—C44—C45—C46	−1.8 (2)
C4—C3—C4A—O1	10.21 (19)	Cl1—C44—C45—C46	177.43 (11)
C2—C3—C4A—O1	−104.62 (16)	C44—C45—C46—C41	−1.1 (2)
C2A—C3—C4A—C5A	−45.66 (16)	C44—C45—C46—Cl2	179.75 (12)
C4—C3—C4A—C5A	−168.03 (12)	C42—C41—C46—C45	3.4 (2)
C2—C3—C4A—C5A	77.15 (15)	C4—C41—C46—C45	−170.57 (13)
N1—C2—C4B—C6B	−57.5 (2)	C42—C41—C46—Cl2	−177.48 (10)
C2B—C2—C4B—C6B	−173.18 (16)	C4—C41—C46—Cl2	8.58 (18)
C3—C2—C4B—C6B	62.5 (2)		

### Hydrogen-bond geometry (Å, °)

**Table:**

*D*—H···*A*	*D*—H	H···*A*	*D*···*A*	*D*—H···*A*
N1B—H1B···O2^i^	0.86	2.02	2.8547 (15)	164

Symmetry codes: (i) −*x*, −*y*, −*z*+1.
